# A multimodal tactile dataset for texture classification on uneven surfaces using a 6-DoF robotic arm

**DOI:** 10.1016/j.dib.2026.113334

**Published:** 2026-10-13

**Authors:** Soheil Khatibi, Frederico Luiz Martins de Sousa, Mateus Nazario Coelho, Vinicius Prado da Fonseca, Thiago Eustaquio Alves de Oliveira

**Affiliations:** a Department of Electrical and Computer Engineering, University of Waterloo, Waterloo, ON, Canada; b Department of Computer Science, Memorial University of Newfoundland, St. John’s, NL, Canada; c Department of Computer Science, Lakehead University, Thunder Bay, ON, Canada; d Department of Computer Science and Media Technology, Linnaeus University, Växjö, G, 352 52, Sweden

**Keywords:** Tactile perception, Tactile sensor, Texture classification, Robot learning, Time series

## Abstract

This article presents a comprehensive tactile dataset designed to facilitate the perception and classification of textures on uneven surfaces. The data was collected using a 6-DoF Lite6 robotic manipulator equipped with a BioIn-Tacto multimodal sensing module. The data acquisition process consists of two main phases. First, an initial probing procedure is conducted to acquire 12 reference points on the curved surface. During this phase, the sensor's Inertial Measurement Unit (IMU) orientation is recorded precisely when the embedded barometer registers contact pressure, accurately capturing the surface normal. Subsequently, the robotic arm performs dynamic exploration by sliding the sensor along the generated trajectory, maintaining consistent contact with the surface. The dataset comprises multi-modal measurements from 12 distinct materials (including various fabrics, wood, leather, and plastics) mounted on curved geometries, with 25 exploratory episodes recorded per texture. The hardware captures nine-degrees-of-freedom Magnetic, Angular Rate, and Gravity (MARG) data alongside deep-pressure readings from a barometer encased in polyurethane rubber. This dataset has substantial reuse potential for researchers developing machine learning models and AI-driven sensorimotor policies for tactile texture classification in realistic environments. Furthermore, as it complements a previous dataset collected with a 4-DoF robot on identical surfaces, it can be used for cross-embodiment research, enabling the community to benchmark sensor performance and evaluate the impact of kinematic constraints on robotic tactile perception.

Specifications Table

**Table:** 

Subject	Robotics
Specific subject area	Tactile texture sensing, perception and recognition.
Type of data	CSV files, python scripts for parsing and pre-processing.
Data collection	Tactile data was acquired using a 6-DoF Lite6 manipulator [1] with a BioIn-Tacto multimodal sensing module [2,3] attached to its end-effector. The module combines a nine-axis Magnetic, Angular Rate, and Gravity (MARG) system with a barometer embedded in polyurethane rubber at the module's base, which registers contact deformation. Mounted on a holder that mimics human exploratory motion, the sensor is slid by the Lite6 along twelve textures bonded to curved surfaces placed on a table, following a trajectory that tracks the surface curvature while maintaining continuous contact. The acquisition protocol replicates that of [4], differing only in the robotic platform. Design details of the BioIn-Tacto module are given in [3]; fabrication details in [2].
Data source location	Institution: Lakehead University City/Town/Region: Orillia, ON Country: Canada
Data accessibility	Repository name: Tactile Data of 12 Textures on Uneven Surfaces Collected with a 6-DoF Robotic Arm Data identification number: 10.17632/yctpcddnm7.2 Direct URL to data: https://data.mendeley.com/datasets/yctpcddnm7/2 Instructions for accessing these data: The full database can be downloaded from the Mendeley Data Repository mentioned above in [5].
Related research article	None

## 1 Value of the Data

• Both this dataset and the presented in [4], provide dynamic tactile signals recorded while the sensor slides over curved substrates, a scenario closer to real-world manipulation than the flat-surface setups common in prior work. The primary distinction between this dataset and that of [4] lies in both the hardware architecture and the data collection methodology. While the robotic platform utilized by [4] features four degrees of freedom (DoF) driven by standard servo motors, the Lite6 manipulator possesses six DoF equipped with high-precision industrial actuators. This fundamental mechanical shift alters the dynamics of the data acquisition process, yielding entirely distinct tactile sensor profiles.
• By providing tactile data from identical textures collected across distinct robotic embodiments, this dataset, when combined with [6], offers the possibility for transfer learning studies. Researchers can utilize this combined data to investigate domain adaptation and the transferability of sensorimotor policies across robots with varying kinematic constraints and actuator fidelities.
• Public availability remains a limitation in tactile perception research, with several prior works releasing partial data or none at all [[7], [8], [9], [10], [11]]. We therefore release this dataset in full, together with its preprocessing scripts, to support reproducibility. Combined with [6], it provides data from the same uneven surfaces collected by two different robots, establishing a valuable baseline for cross-embodiment research. It allows researchers to benchmark sensor performance across robots with varying kinematic limitations, offering critical insights into how hardware constraints affect tactile data quality.

## 2 Background

This dataset offers tactile (MARG and barometric) data recordings for texture recognition gathered while a sensor slides over uneven surfaces in a concave convex shape, acquired with a Lite6 robotic arm equipped with a BioIn-Tacto sensing module. It directly extends the dataset presented in [4]. Both share the same sensor, the same twelve textures, and the same collection protocol, differing only in the manipulator, a 6-DoF Lite6 with industrial actuators replaces the 4-DoF servo-driven OpenManipulator-X. This pairing makes the two datasets a controlled testbed for cross-embodiment studies, in which the influence of the robot platform on tactile representations can be isolated. Beyond machine learning for texture classification in realistic, uneven-surface conditions, the dataset supports research on transfer learning, domain adaptation, and few-/zero-shot and sequential learning across manipulators, and further increases the availability of public tactile data.

## 3 Data Description

The dataset comprises raw tactile sensor measurements stored in CSV format, organized within a specific hierarchical folder structure. No TF data from the robot is available at this moment. Each directory contains multiple readings corresponding to distinct textures and exploratory episodes. Additionally, the dataset provides sample Python scripts that utilize the Pandas library to parse and access the data efficiently. Table 1 outlines the complete directory structure and subfolder descriptions.

**Table 1:** Folder structure of the dataset and description of each processing stage.

*Folder Name*	*Description*
**Data/**	Main folder containing all data subfolders.
**Data/0_Raw/**	Raw CSVs extracted from ROS bags, organized by shape/texture (T1–T12)/experiment (25 movements per texture).
**Data/1_Merged/**	Per-sensor CSVs merged into one time-aligned CSV per experiment.
**Data/2_Trimmed/**	Recordings trimmed to keep only the valid interaction segment.
**Data/3_Normalized/**	Trimmed signals normalized per experiment (normalized_data.csv).
**Data/4_Windowed/**	Signals split into sliding windows of 128, 256, and 512 samples.
**Data/5_Reindexed/**	Windows reindexed and merged for consistent numbering across experiments.
**Data/6_NPY/**	Final .npy arrays per dataset and window size, plus windows_per_experiment.json metadata.
**Data/7_Folded/**	Windows organized into cross-validation folds for train/test evaluation.
**Scripts/**	Contains all Python scripts for data preprocessing.
**ROS_Bags/**	Contains the ROS bags from the 12 textures, with each probing and data collection phase.
**STL/**	Contains the concave convex shape .stl file.

The **/Data/** folder is organized into eight sequential stages, **0_Raw** through **7_Folded**, reflecting the data processing pipeline. The **0_Raw** folder establishes the base hierarchy adopted throughout the dataset: a surface category folder (*concave_convex*), containing twelve texture folders (*T1 to T12*), each with twenty-five episode subfolders (*1 to 25*) that hold the raw sensor recordings (*m_baros_serial.csv* and *m_imus_bias_removed.csv*). T*he m_imus_bias_removed.csv* files contain the closest available representation of the unprocessed inertial stream. They are produced by a ROS2 node (*middle_imu_bias_removed*) that subscribes to the raw serial IMU packets and republishes them after startup calibration only. Before any data are forwarded, the node performs a one-time, stationary calibration sequence: gyroscope offsets are estimated from a 10 s stationary window via a dedicated service, a residual Z-axis gyroscope trim is refined over 300 stationary samples, and accelerometer offsets are estimated over 300 stationary samples against the expected gravity vector. Incoming packets are dropped until all three calibration stages complete, and safety thresholds flag abnormally large biases. The calibration ensures that the recorded streams are free of static sensor offsets while remaining otherwise unprocessed.

The intermediate stages (**1_Merged** to **5_Reindexed**) preserve this same surface/texture/episode hierarchy, differing only in the files stored at the episode level: each stage replaces the previous output with its processed counterpart, namely the merged (*merged_data.csv*), trimmed (*trimmed_data.csv*), and normalized (*normalized_data.csv*) signals, followed by fixed-size window segments (*window_1.csv to window_N.csv, organized into windows/128, windows/256, and windows/512 subfolders*) in the windowed and reindexed stages. Each normalized episode is segmented into fixed-length windows of 128, 256, and 512 samples, producing three parallel representations of the same recording. Windows are extracted with a stride equal to half the window size (i.e., 50% overlap), so consecutive windows share half of their samples. At the acquisition sampling rate of approximately 130 Hz, the three window sizes correspond to temporal spans of roughly [0.98, 1.97, and 3.94] s, respectively, capturing texture signatures at short, medium, and long temporal scales. The exact number of windows generated per episode and per texture is reported in the *windows_per_experiment.json* metadata files. Because overlapping windows from the same episode share content, all windows originating from one episode are assigned to the same cross-validation fold, as detailed in the fold-construction description (**7_Folded**), preventing correlated samples from appearing in both training and test sets.

In the trimming stage, recordings are trimmed automatically to isolate the valid interaction segment, using the barometric channel as the contact indicator. For each episode, a pressure baseline is first computed as the median of the initial 100 samples, when the sensor is not yet in contact. All trim points are stored in a machine-readable file (*trim_points.json*), which can be manually audited and edited. Re-running the pipeline with *–use_json* applies the curated indices verbatim, ensuring that the trimming stage is fully deterministic and reproducible. Optional preview plots of each trim decision can be generated for visual verification.

Each trimmed episode is normalized independently using per-channel min–max scaling. For every episode, each of the ten signal channels (barometric level; acceleration ax–az; angular velocity gx–gz; and magnetic field mx–mz) is rescaled to the [0, 1] interval as

$$x^{\prime }=\left(x-x\;\text{min}\right)/\left(x\;\text{max}=x\;\text{min}\right),$$

where x_min and x_max are the minimum and maximum values of that channel within the episode. All other columns, including timestamps, are preserved unchanged. Normalization is deliberately computed per episode rather than globally. The scaling parameters of one episode never use information from any other episode, so no statistics can leak across the train/test boundary as long as episodes are kept in a single fold.

The final two stages depart from this hierarchy: **6_NPY** consolidates each window size into ready-to-use NumPy arrays (X_windows_*.npy and y_labels_*.npy) together with JSON metadata files, and **7_Folded** reorganizes the windows into cross-validation splits (*Lite6-128/256/512*, each with *2-Fold, 4-Fold, and 5-Fold* strategies) containing per-episode fold files (*fold_K_x.npy and fold_K_y.npy*).

Here is the hierarchy of each folder inside **/Data/**:

 ● **/Data/0_Raw/**.

 ° **Folder:** concave_convex (surface/shape category).

 ° **Subfolder**: T1 to T12 (each representing a texture).

 ■ **Subfolder**: 1 to 25 (each representing an exploratory episode).

 ● **CSV file**: m_baros_serial.csv (contains barometric data).

 ● **CSV file**: m_imus_bias_removed.csv (contains MARG data).

● **/Data/1_Merged/**.

 ° **Folder**: concave_convex → T1–T12 → 1–25.

 ■ **CSV file**: merged_data.csv (time-aligned barometric and MARG data).

● **/Data/2_Trimmed/**.

 ° **Folder**: concave_convex → T1–T12 → 1–25.

 ■ **CSV file**: trimmed_data.csv (recording trimmed to the valid interaction segment).

● **/Data/3_Normalized/**.

 ° **Folder**: concave_convex → T1–T12 → 1–25.

 ■ **CSV file:** normalized_data.csv (normalized signals).

● **/Data/4_Windowed/**.

 ° **Folder:** concave_convex → T1–T12 → 1–25.

 ■ **Subfolder**: windows/128, windows/256, windows/512 (one per window size).

 ° **CSV file**: window_1.csv to window_N.csv (fixed-size signal segments).

● **/Data/5_Reindexed/**.

 ° **Folders**: concave_convex → T1–T12 → 1–25.

 ■ **Subfolder:** windows/128, windows/256, windows/512.

 ● **CSV files:** window_1.csv to window_N.csv (renumbered sequentially).

● **/Data/6_NPY/**.

 ° **NPY files:** X_windows_128.npy, X_windows_256.npy, X_windows_512.npy (windowed signal arrays).

 ° **NPY files:** y_labels_128.npy, y_labels_256.npy, y_labels_512.npy (corresponding texture labels).

 ° **JSON files:** windows_per_experiment.json and windows_per_experiment_lite6.json (window counts per texture and episode).

● **/Data/7_Folded/**.

 ° **Folder:** Lite6-128, Lite6-256, Lite6-512 (one per window size).

 ■ **Subfolder:** 2-Fold, 4-Fold, 5-Fold (cross-validation split strategies).

 ● **Subfolder:** episode folders (1–50 for 2-Fold, 1–25 for 4-Fold, 1–20 for 5-Fold).

 ° **NPY files:** fold_K_x.npy and fold_K_y.npy for K = 1..N (windows and labels assigned to each fold).

**Scripts/**.

The **Scripts/** folder contains the Python and Shell scripts used to automate the data preprocessing pipeline:

Main scripts (run by **run.sh**, in order):

  • **merge_datasets.sh:** Runs merge.py per shape to build 1_Merged.

  • **trim_dataset.sh / trim.py:** Trims recordings; also supports –create_json_only / –use_json modes and per-texture or preview runs.

  • **normalize_datasets.sh:** Runs normalize.py on every trimmed CSV to build 3_Normalized.

  • **create_windows.sh:** Runs window_creator.py per dataset/surface to generate the 128/256/512-sample windows.

  • **reindex_windows.sh:** Runs reindex_windows.py to reindex and merge windows into 6_Reindexed.

  • **create_npy.sh:** Runs npy_creator.py per dataset and window size to export .npy files.

  • **generate_windows_metadata.sh:** Runs generate_windows_metadata.py to write per-dataset windows_per_experiment.json files.

The pipeline thus converts raw barometric and MARG recordings into analysis-ready arrays, including cross-validation splits. Example rows from the two raw sensor files are shown in Table 2, Table 3.

**Table 2:** First five rows of *m_baros_serial.csv* (**0_Raw/T1/1**), showing the raw ADC barometric readings in *field.baros0.baro_level*.

*%time*	*field.baros0.baro_level*
1752861711982599326	633.0
1752861711985758131	633.0
1752861711992456603	634.0
1752861712000006370	633.0
1752861712006327350	633.0

**Table 3:** First five rows of *m_imus_bias_removed.csv* (**0_Raw/T1/1**), showing MARG readings after bias removal: linear acceleration (ax–az, m/s²), angular velocity (gx–gz, rad/s), and magnetic field (mx–mz, gauss) in the sensor reference frame. The time column is shown as Unix epoch nanoseconds.

*%time*	*field.imus0* *.ax*	*field.imus0* *.ay*	*field.imus0* *.az*	*field.imus0* *.gx*	*field.imus0* *.gy*	*field.imus0* *.gz*	*field.imus0* *.mx*	*field.imus0* *.my*	*field.imus0* *.mz*
1752861711983102211	−1.688510775566101	5.435436248779297	7.539340972900391	0.0184304341673851	0.01755504682660103	−0.017330117523670197	−0.0341796875	0.154541015625	0.1256103515625
1752861711992768214	−1.688510775566101	5.435436248779297	7.539340972900391	−0.04068375378847122	−0.012328281998634338	0.009682323783636093	−0.0341796875	0.154541015625	0.1256103515625
1752861712000317992	−1.8590975999832153	5.613804340362549	7.495646953582764	0.02156231179833412	−0.012980757281184196	0.03160546347498894	−0.033447265625	0.15472412109375	0.13128662109375
1752861712006883253	−1.8590975999832153	5.613804340362549	7.495646953582764	0.02156231179833412	−0.012980757281184196	0.03160546347498894	−0.033447265625	0.15472412109375	0.13128662109375
1752861712013402799	−1.8590975999832153	5.613804340362549	7.495646953582764	−0.0024487487971782684	0.0022871457040309906	−0.017460614442825317	−0.033447265625	0.17034912109375	0.13885498046875

## 4 Experimental Design, Materials and Methods

Data collection was performed with a 6-DoF Lite6 manipulator carrying the BioIn-Tacto sensing module on its end-effector, sliding it across 12 texturess. All textures were bonded to 3D-printed concave–convex substrates of 5 cm width, 12 cm length with a varying height, providing a fixed curved geometry that the exploration trajectory follows. The exploration trajectory for each surface is generated from an initial probing phase, the robot making contact with the surface at twelve reference points, recording the IMU orientation at the instant the barometer detects contact pressure, so that the measured inclination closely approximates the local surface normal. These normals drive the trajectory generation [12], yielding sliding paths that follow the surface geometry with consistent contact throughout. Fig. 1 illustrates the probing phase, while the Table 4 contains its parameters. Fig. 2 illustrates the data collection setup while Table 5 contains its parameters. A Y supplementary video illustrating both the probing phase and the dynamic exploration is available at https://youtu.be/kiv4AitUa-I.

**Fig. 1:**
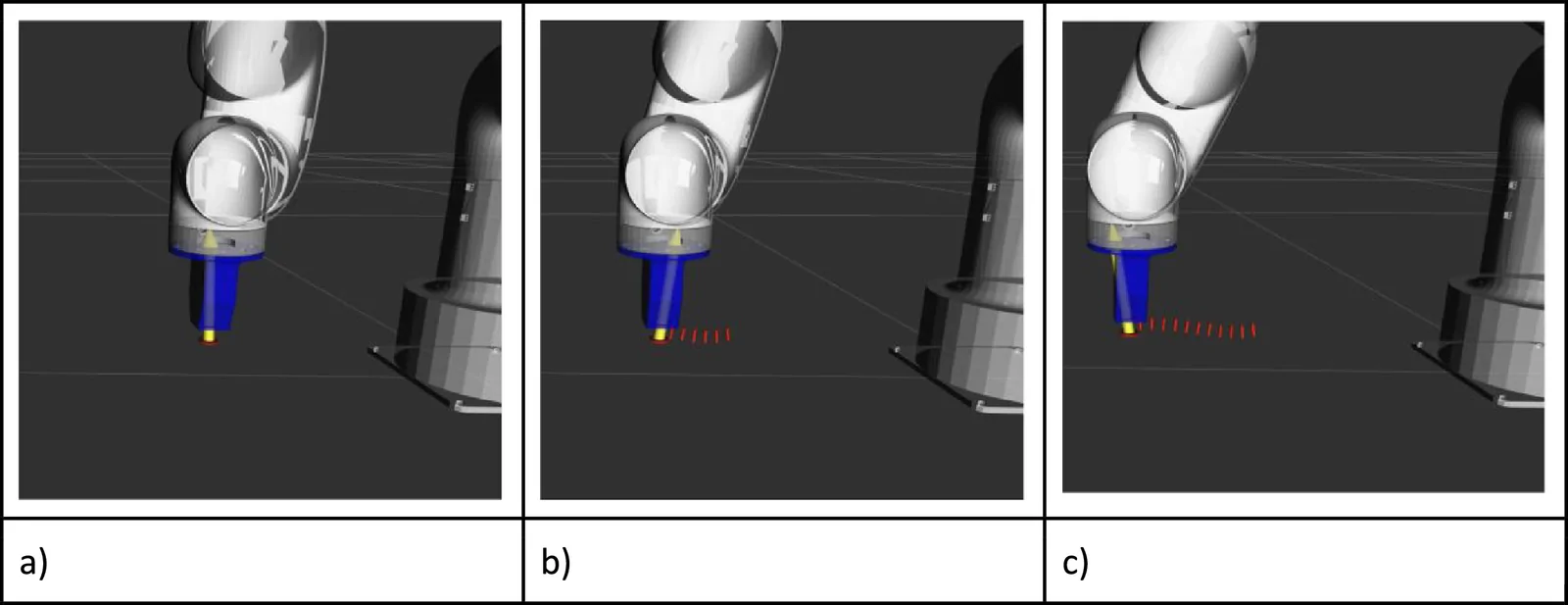
Overview of the probing phase, collecting the normal points on an uneven surface with a textured material: a) The robot at the first probing point; b) The robot at the seventh point of the surface; c) The robot at the last point of the surface. The yellow area displays the normal vector inclination of the point.

**Table 4:** Parameters of the probing phase for the reconstruction of the surface and material.

Parameter	Value	Description
Number of probing points	12	Reference contacts used to estimate surface normals
First probing position (x)	0.235 m	Start of the probing line in the robot base frame
Step between points (Δx)	0.01 m	Spacing between consecutive probing points (total span: 0.11 m)
Hover height (z)	0.05 m	Height above the surface before each probe
Drop distance	0.025 m	Vertical descent from hover to contact
Contact trigger threshold	450 ADC counts	Barometric level indicating contact; IMU orientation recorded at this instant
Velocity scaling	0.10	Fraction of the manipulator's maximum velocity (MoveIt)
Acceleration scaling	0.10	Fraction of the manipulator's maximum acceleration (MoveIt)

**Fig. 2:**
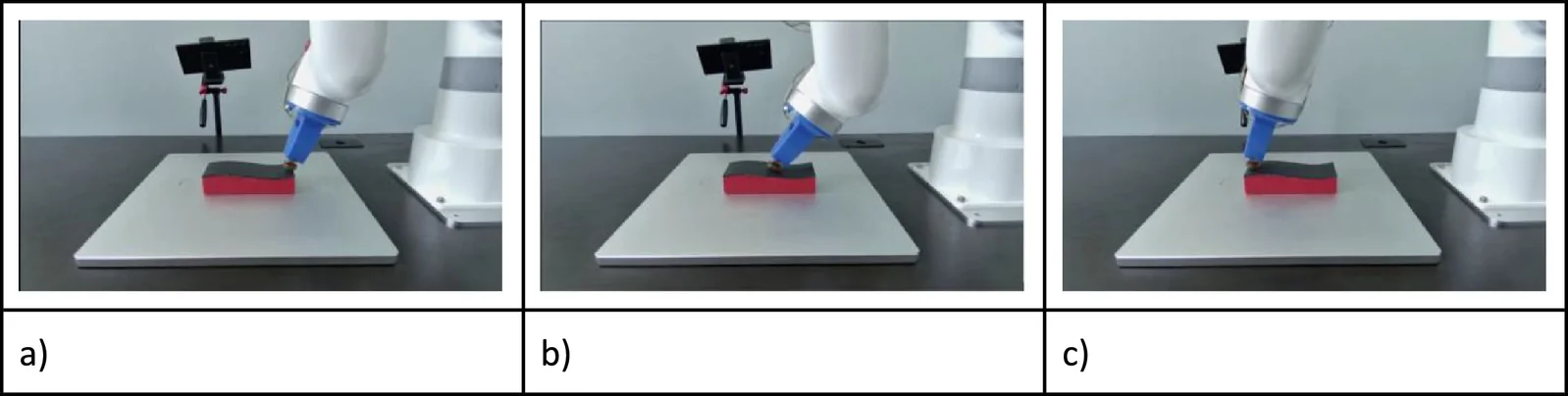
Data collection of the Lite6 with BioIn-Tacto over a curved textured surface: start (a), middle (b), and end (c) of the exploration path.

**Table 5:** Parameters of the exploration phase for the data collection of the surface and material with BioIn-Tacto.

Parameter	Value	Description
Episodes per texture	25	Repeated sliding explorations per texture in a single session
Contact offset (shift_z)	0.008 m	Vertical offset maintaining sensor–surface contact during sliding
Velocity scaling — sliding	0.20	MoveIt velocity factor during texture exploration
Acceleration scaling — sliding	0.20	MoveIt acceleration factor during texture exploration
Home / pre-probing pose	(0.22, 0.0, 0.05) m, (180°, −45°, 0°)	Starting configuration (x, y, z; roll, pitch, yaw)
Release pose	(0.35, 0.0, 0.05) m, (180°, −45°, 0°)	End-of-episode retract configuration

The textures explored in this dataset consisted of brocade fabric, carpet wool, embossed plastic, honeycomb fabric, mesh cotton, mesh leather, open weave cotton, reptile-patterned leather, ridged polymer, silicone mesh, tight weave cotton, and wood. Fig. 3 shows the texture set.

**Fig. 3:**
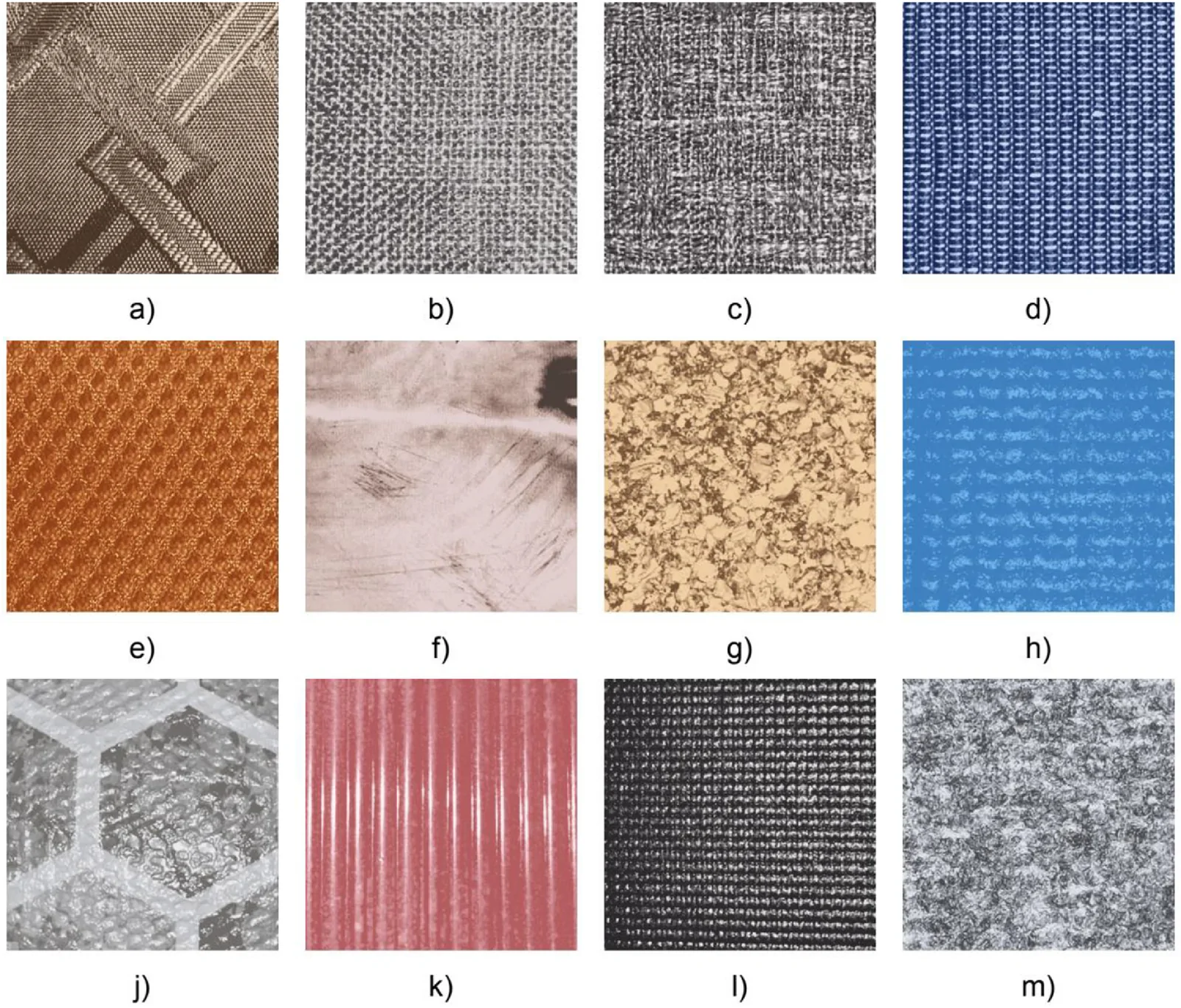
The 12 textures used in the experiments, extracted from [4]: a) brocade fabric; b) open weave cotton; c) tight weave cotton; d) mesh cotton; e) honeycomb fabric; f) embossed plastic; g) wood; h) silicone mesh; i) reptile-patterned leather; j) ridged polymer; k) mesh leather; l) carpet wool.

Throughout each slide, the module logged MARG signals (acceleration, angular rate, magnetic field) while the embedded barometer recorded the deformation-induced pressure response The magnetometer channels are released as part of the complete MARG stream. They are used in the pipelines that estimate sensor orientation from the full nine-axis measurement, and they preserve the module's entire output for future studies. In this work, all channels are provided to classification models jointly, without isolating individual modalities (or studying their influence itself). Fig. 4, Fig. 5 show example traces.

**Fig. 4:**
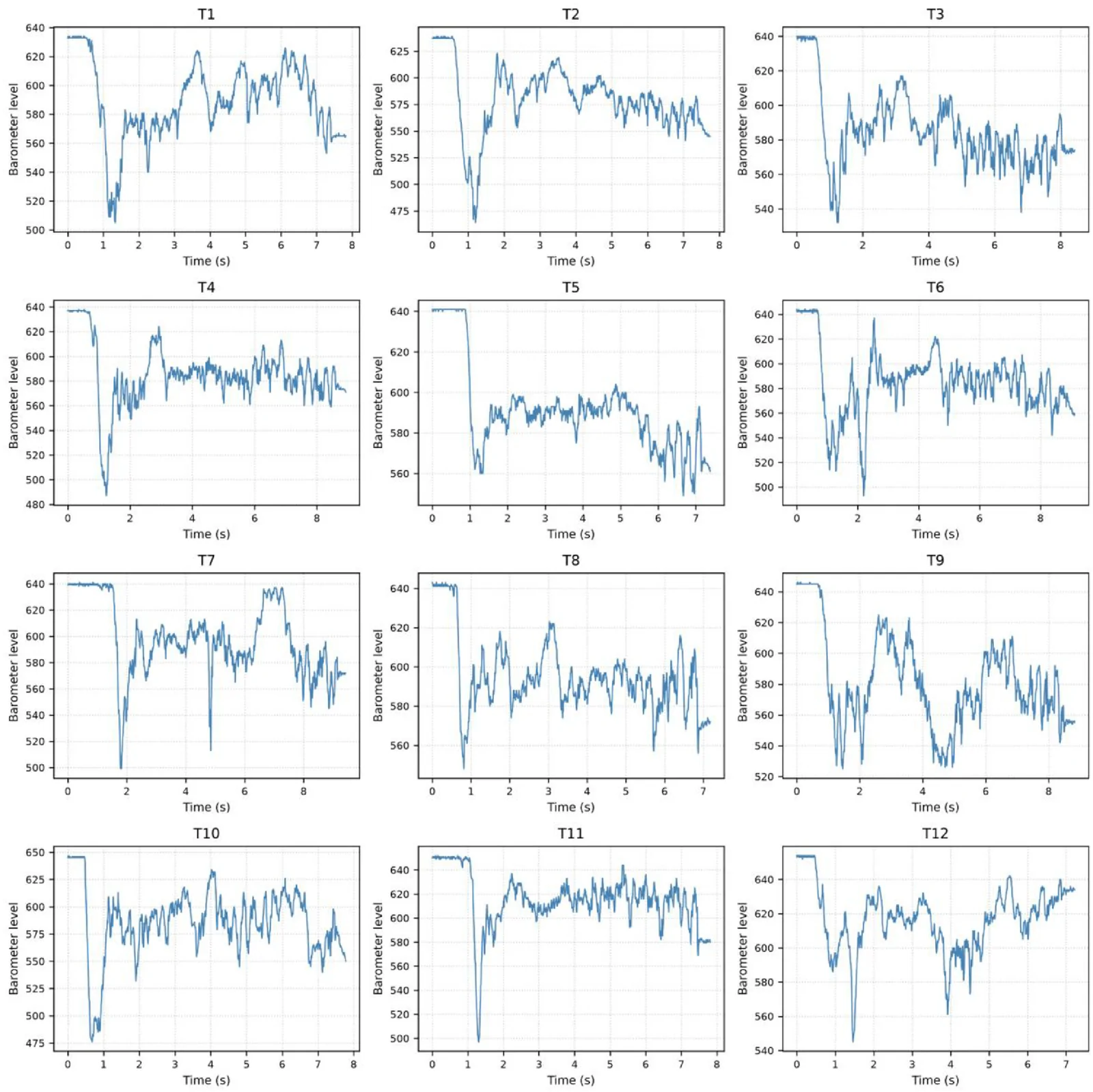
Barometric data for the twelve textures recorded with the Lite6 setup.

**Fig. 5:**
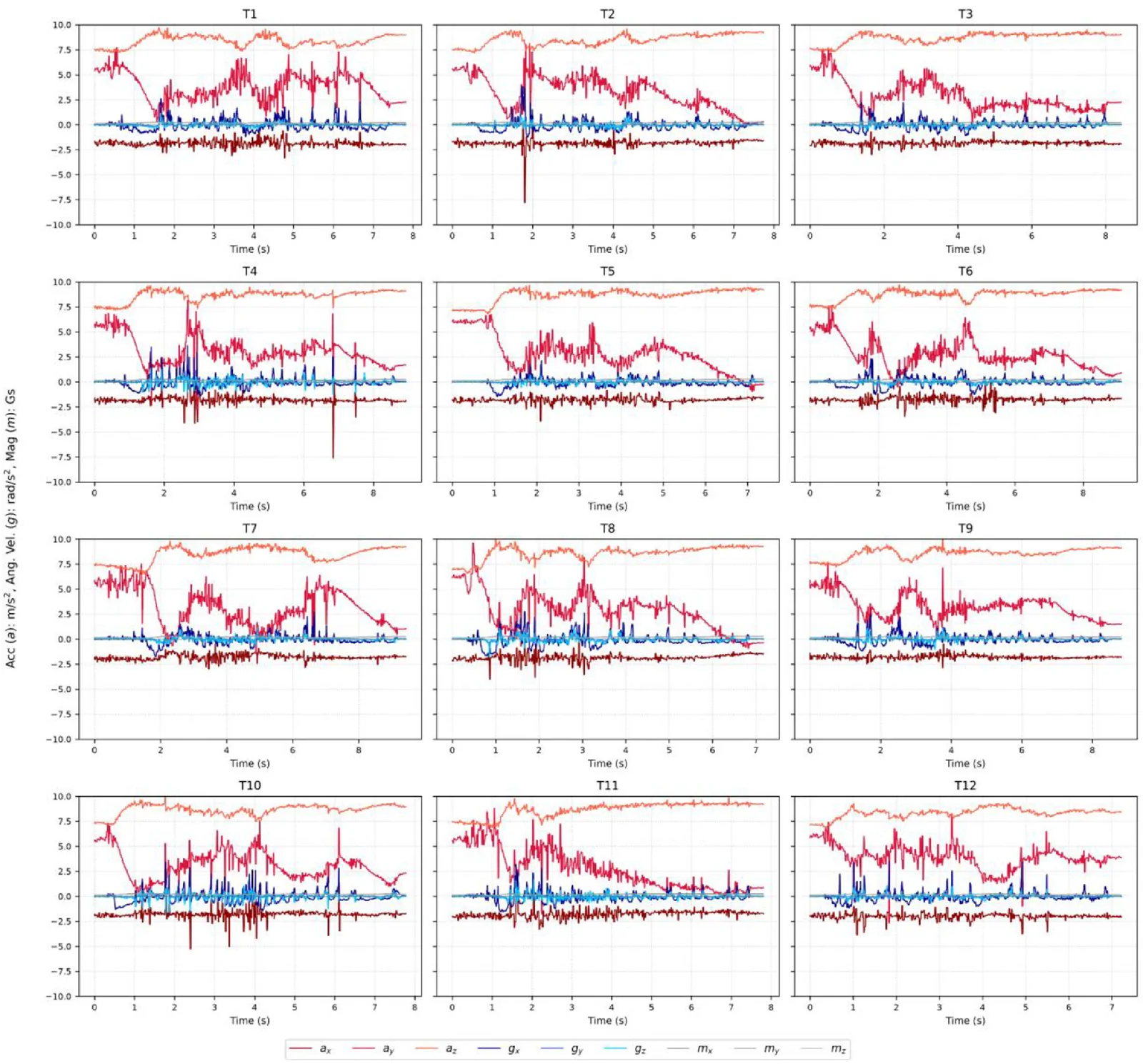
MARG data for the twelve textures recorded with the Lite6 setup.

## Limitations

This dataset is bounded by its acquisition design, where all data come from a single sensing architecture (BioIn-Tacto) on one manipulator (Lite6), covering twelve textures explored in twenty-five episodes each on laboratory fabricated curved geometries, using a fixed sliding velocity and a predefined trajectory with a compliant holder, so the range of contact forces, speeds, and surface conditions represented is narrow, and results do not directly generalize to other sensors, robots, or surfaces without further validation.

## CRediT Author Statement

**Soheil Khatibi:** Data collection, Validation, Data curation, Visualization, and Software. **Frederico Luiz Martins de Sousa**: Writing- Reviewing and Editing, Software, Data curation. **Mateus Nazario Coelho**: Writing- Reviewing and Editing, Software. **Vinicius Prado da Fonseca:** Supervision, Conceptualization, Writing- Reviewing and Editing, Validation and Funding acquisition. **Thiago Eustaquio Alves de Oliveira**, Supervision, Conceptualization, Writing- Reviewing and Editing, Validation, and Funding acquisition.

## Ethics Statement

This research focused exclusively on the technical evaluation of a tactile-enabled robotic manipulator and the BioIn-Tacto sensing module. No human subjects, animal experiments, or social media data were involved in this study. Consequently, ethical committee approval, informed consent, and data redistribution policies are not applicable. The study fully complies with the ethical guidelines outlined in the journal's Guide for Authors.

## Declaration of Generative AI and AI-assisted Technologies in the Manuscript Preparation Process

During the preparation of this work, the authors used Google Gemini for improving the English language and for better readability of this manuscript. The authors reviewed and edited the output as needed and take full responsibility for the content of the published article.

## Appendix Supplementary materials

Image, video 1 dummy alt text
